# Structural characterisation of inhibitory and non-inhibitory MMP-9–TIMP-1 complexes and implications for regulatory mechanisms of MMP-9

**DOI:** 10.1038/s41598-021-92881-x

**Published:** 2021-06-28

**Authors:** Łukasz Charzewski, Krystiana A. Krzyśko, Bogdan Lesyng

**Affiliations:** Department of Biophysics, Faculty of Physics, University of Warsaw, Pasteura 5, 02-093 Warsaw, Poland

**Keywords:** Computational biology and bioinformatics, Structural biology, Molecular medicine

## Abstract

MMP-9 plays a number of important physiological functions but is also responsible for many pathological processes, including cancer invasion, metastasis, and angiogenesis. It is, therefore, crucial to understand its enzymatic activity, including activation and inhibition mechanisms. This enzyme may also be partially involved in the “cytokine storm” that is characteristic of COVID-19 disease (SARS-CoV-2), as well as in the molecular mechanisms responsible for lung fibrosis. Due to the variety of processing pathways involving MMP-9 in biological systems and its uniqueness due to the O-glycosylated domain (OGD) and fibronectin-like (FBN) domain, specific interactions with its natural TIMP-1 inhibitor should be carefully studied, because they differ significantly from other homologous systems. In particular, earlier experimental studies have indicated that the newly characterised circular form of a proMMP-9 homotrimer exhibits stronger binding properties to TIMP-1 compared to its monomeric form. However, molecular structures of the complexes and the binding mechanisms remain unknown. The purpose of this study is to fill in the gaps in knowledge. Molecular modelling methods are applied to build the inhibitory and non-inhibitory MMP-9–TIMP-1 complexes, which allows for a detailed description of these structures and should allow for a better understanding of the regulatory processes in which MMP-9 is involved.

## Introduction

Matrix metalloproteinase 9 (MMP-9) is a secreted protein exhibiting endoproteolytic activity^1^. It is secreted in a latent form (proMMP-9) containing a propeptide (PRO) in its catalytic site, which upon activation is cleaved or destabilised^2^. MMP-9 is engaged in the degradation of extracellular matrix components, including degradation of type I^1^ and type III-V collagens, elastin, aggrecan, and laminins^3^—one should note, however, that its biochemical function is much broader. MMP-9 works as an activator, or activity enhancer, of signalling molecules like cytokines or chemokines, such as pro-IL-1β and IL-8. MMP-9 is found in neutrophil tertiary granules^4^, and its activity plays a significant role in the “cytokine storm” syndrome^5^, which is one of the contributors to COVID-19 severity and mortality^6^. MMP-9 overactivation is also associated with tissue damage during inflammation^7^. In contrast, molecules like CTAP-III, PF-4, and GRO-α can be inactivated by MMP-9. The catalytic activity of MMP-9 is inhibited by proteins belonging to the tissue inhibitors of the metalloproteinase (TIMP) family. Each of the four members exhibits inhibitory potential for MMP-9 but with different affinities^8,9^. MMPs and TIMPs, including MMP-9 and TIMP-1, are particularly involved in the pathological molecular mechanisms of lung fibrosis^10,11^. TIMPs bind MMPs in a 1:1 ratio; however, by interacting with more than one binding site on MMPs, they can bind either to the catalytic domain (CAT), forming an inhibitory complex, or to the hemopexin domain (HPX), forming a non-inhibitory one^12^. Interestingly, MMP-9 is very often secreted from the cell in the form of a non-inhibitory TIMP-1 complex^13^. Out of all MMPs, only two (MMP-9 and MMP-2) possess an additional insert, called the fibronectin-like domain (FBN). FBN function in the TIMPs binding process has been poorly investigated. So far, it has been established that it plays a minor role in the TIMP binding. One should note that the experiments were carried out using the activated form of MMP-2, including HPX, which could influence the association constant. The truncated forms of TIMPs were applied to overcome this obstacle^14^, but it is unclear whether this manipulation itself influenced the binding contacts with FBN.

MMP-9 is secreted from the cell as a mixture of monomers and oligomers, with a more abundant monomeric form. Electrophoretic analysis indicates the mass of oligomers composed of 2 or 3 subunits, although their exact number remains unclear^15^. Crystallographic studies showed that MMP-9 HPX can dimerise due to hydrophobic interactions formed between their IV beta-propellers blades^16^. This finding was inconsistent with an observation that their subunits dissociate under reducing conditions, which implies cysteine involvement in the oligomer formation^17^. However, one should note that an oligomer of a Cys468Ala MMP-9 mutant, sensitive to reduction conditions, was also isolated^18^.

There are two cysteine residues, which are considered to be responsible for the oligomerisation process. Those are Cys468, positioned halfway in the O-glycosylated domain (OGD), and Cys674, located in an IV blade of HPX. Accessibility to Cys674 is very limited because it is buried in a hydrophobic environment. This probably led to a tendency to interpret oligomers as dimers rather than trimers^19^. Other cysteine residues are unlikely to be involved in oligomerisation because they either form well-documented disulphide bridges or belong to a cysteine-switch motif.

In 2015, applying AFM and TEM methods, the homotrimeric proMMP-9 structure was proposed and visualised^15^. The trimer turned out to be unstable under reducing conditions. Such behaviour indicates the involvement of disulphide bridging in the complex formation. Molecular image measurements revealed two distinct populations, which share an overall circular shape, but with different dimensions. These variants are in a ratio of 3:1, where the smaller complexes are more abundant. The inner and outer diameters in the TEM photos indicate the free space between the subunits and also estimate their outer dimensions. The inner and outer diameters are 2.8 ± 0.7 nm and 10.8 ± 2.1 nm, respectively, for the smaller complex, and 6.9 ± 0.8 nm and 14.1 ± 2.2 nm for the larger one. These dimensions and the shape analysis indicated one conformer with the subunit arrangement mainly stabilised by the Cys468-Cys674 bridge but also by non-covalent interactions between HPX and CAT/FBN. The TEM images also revealed that OGD is directed into the interior of the circular structure. The experiments also clarified that remaining in a monomeric state or forming homotrimers is not a consequence of differences in the glycosylation pattern^15^. Interestingly, the strong binding of TIMP-1 to such structures does not increase the complexes’ observed dimensions. Until now, the precise location of TIMP-1 has not been established, but the contacts with the catalytic^20^ and hemopexin^21^ MMP domains are well described for smaller systems. The TIMP-1 molecule is a rigid lobe; its two domains are connected with a disulphide bond and form tight contacts^20^. Structural experiments carried out on TIMP-2 revealed that upon binding to the MMP CAT, a slight rotation (approx. 13°) between its domains takes place^21^. The TIMPs unstructured C-terminus forms a kind of a flexible tail, which interacts with HPX. In the MMP-2 /TIMP-2 complex, it penetrates the space between the III and IV beta-propeller blades creating three salt bridges. In TIMPs 2–4, this tail is 12 amino acids long, while in TIMP-1, it is composed only of three residues.

This work aims to study molecular interactions leading to homotrimerisation of MMP-9, and to design structural models describing the MMP-9–TIMP-1 binding process—leading to two distinct MMP-9 forms: a terminal-inhibitory complex and a circular heterohexamer. Such knowledge is required for a better understanding of the MMP-9 kinetics and may be helpful in designing its selective activity inhibitors in a given molecular state.

## Materials and methods

### Modelling of inhibitory and non-inhibitory complexes

A preliminary inhibitory complex model was developed using the MMP-3–TIMP-1 complex (PDB ID: 1UEA) as the template^20^. The C-terminally truncated proMMP-9 structure (PDB ID: 1L6J)^22^, after PRO removal, was structurally aligned with the MMP-3 structure, and all homological interactions were adjusted manually^20^. After energy minimisation (EM) of the resulting complex, 5 ns MD simulations were applied.

A partial non-inhibitory complex model of MMP-9 HPX with TIMP-1 was built using the structure of a homological proMMP-2–TIMP-2 complex from 1GXD PDB entry (chains A/C)^23^. The HPX structure of 1ITV (chain A)^16^ and the TIMP-1 structure of 1UEA^20^ PDB entries were used to build the complex. Missing TIMP-1 C-terminal residues were appended. Interactions similar to those found in the template complex were modelled, except for the TIMP-1 C-terminal flexible tail. Conformations of six C-terminal residues were determined with LowModeMD^24^ implemented in the MOE 2016.08 modelling environment. One thousand iterations were run applying the CHARMM27 force field. Regarding EM, the RMS of the energy gradient was set to 0.1 kcal/mol. Structures characterised by the total energy up to 100 kcal/mol above the minimum were used in 5 ns MD simulations.

### Modelling of terminal inhibitory complexes

The terminal-inhibitory MMP-9–TIMP-1 complex model was built by aligning the partial-inhibitory complex with the non-inhibitory one. Contacts known from the partial-inhibitory complex were remodelled, except for those that could affect the stability of TIMP-1 interactions with HPX. Also, new contacts between HPX and FBN were found and adjusted. A missing OGD was included in the system as an extended, unstructured loop and pre-optimised. The complex was subjected to 150 ns MD.

### Modelling of circular complex modelling

The proMMP-9 homotrimeric structure is determined by two nonoverlapping interfaces between CAT/FBN and HPX. In order to predict possible contact sites between them, the CAT/FBN structure (PDB ID: 1L6J)^22^ and the HPX structure (PDB ID: 1ITV, the chain A)^16^ were used. Rigid protein–protein docking was carried out using the GRAMM-X server^25,26^, where HPX was docked to CAT/FBN. Results that potentially corresponded to the homotrimeric model were divided into two groups: one with HPX forming an interface with FBN and the second with HPX being in contact with CAT. All possible combinations between those groups were tested by applying structural alignment five times, resulting in full, homotrimeric models, which were evaluated by their overall shape and occurrence of significant steric clashes. The best-constructed structure was chosen for further research. The first and the last domain were slightly rotated to obtain higher symmetry of the homotrimer. Such a model was subjected to several 10 ns MD simulations. TIMP-1 molecules were then added to the homotrimeric structure using the non-inhibitory MMP-9–TIMP-1 complex structure. After modelling of previously identified interactions with HPX, new possible interactions between TIMP-1 and the proMMP-9 PRO/CAT were found and adjusted. TIMP-1 C-terminus conformation was determined using LowModeMD, and the resulting complexes were refined using MD and applying the same conditions as previously.

### Calculation details

Each MD simulation was executed applying the NAMD2.12 modelling environment^27^ and CHARMM27 force field^28^ in a periodic cuboid cell. Water molecules were represented with the TIP3 model. Explicit ions with concentration corresponding to 0.05 M ionic strength were used. Atmospheric pressure and a temperature of 310 K were applied.

Structural stability was confirmed by applying the RMSD and internal energy analysis on the MD trajectories. The interaction energies between the TIMP-1 and MMP-9 structures were computed using the MM-GBSA^29^ method, applying trajectory snapshots after systems equilibration and stabilisation. The internal energy of the TIMP-1 C-terminus was computed as the conformational energy of the six C-terminal residues. The contact area between the complex subunits was established using SASA calculations on the MD trajectories. For every frame, the difference between subunits areas was computed and averaged.

## Results and discussion

### Model of the MMP-9–TIMP-1 complex and its biological implications

Structural data of the activated MMP-3 CAT^20^, the TIMP-1 inhibiting complex, the proMMP-2 HPX, and the TIMP-2 non-inhibiting complex^23^ were analysed. This knowledge allowed us to construct homological complexes, including CAT/FBN and HPX of MMP-9 with its inhibitor—TIMP-1.

Because there was no accurate information on the relative orientation of the MMP-9 domains, nor on the influence of the inhibitor binding on the spatial MMP-9 structure, the complexes of both domains with TIMP-1 were modelled separately. The modelled complex structures appeared to be stable during the MD simulations. The interaction energy of the MMP-9 CAT/FBN with TIMP-1 is − 67.77 ± 0.33 kcal/mol [mean ± SEM], which is lower than in the case of the reference MMP-3–TIMP-1 system (− 63.89 ± 0.19 kcal/mol). FBN, not present in the reference system, creates several contacts lowering the interaction energy (Table 1). The energy value computed only between MMP-9 CAT and TIMP-1 (− 57.41 ± 0.29 kcal/mol) is higher than in the reference system. This results from the lack of three interactions present in the complex containing MMP-3. Ser134 of TIMP-1 forms hydrogen bonds with MMP-3 Gly192 and Thr190. Also, TIMP-1 Pro136 forms a hydrophobic contact with MMP-3 Thr191. Those interactions are absent in the system containing MMP-9 because FBN is inserted in this particular place. In the non-inhibitory MMP-9–TIMP-1 complex, the interaction energy (− 43.54 ± 0.18 kcal/mol) is similar to that of the reference MMP-2–TIMP-2 complex (− 45.68 ± 0.21 kcal/mol) when comparing the systems without the TIMPs C-termini. In these systems, including however the C-terminus, the interaction energies are -62.60 ± 0.13 kcal/mol and − 87.95 ± 0.30 kcal/mol, respectively. The mean interaction energy in the MMP-9 hemopexin–TIMP-1 complex model is higher than in the reference system due to the longer TIMP-2 C-terminus. The energy values computed without additional amino acids are comparable to the ones in our model. These results suggest that the models were prepared correctly. A list of specific interactions found in the model systems is presented in Table 1. Similarly to the references, in the MMP-9–TIMP-1 complex most of the interactions are hydrophobic. In the inhibitory complex, the N-terminus of TIMP-1 is inserted into the MMP-9 cleft forming contacts with the catalytic zinc ion. These findings effectively reproduce contacts determined in crystallography experiments^20,23^. The interface surface areas between the MMP-9–TIMP-1 complex subunits are 1426.45 ± 2.40 Å^2^ for the MMP-9 CAT/FBN, and 954.87 ± 0.49 Å^2^ for the MMP-9 HPX. These are comparable to references: 1277.50 ± 1.59 Å^2^ and 1253.53 ± 1.88 Å^2^, respectively. A few amino acids interacting in the complexes are highly conserved. For TIMP-1, they are Cys1, Cys3, Pro5, Val29, Pro136, Leu146, and Trp176. Additionally, the hydrophobic properties of TIMP-1 amino acids interacting with them are preserved in TIMPs: Ala65, Val69, Ile135, Leu139, Leu151, and Leu164^30^. In MMP-9, the interacting conserved amino acids are Pro180, Leu188, Glu402, Tyr423, and Cys388, which belong to FBN. Amino acids with preserved properties in MMPs are Phe192, Pro655, and Trp680^31^. The model of the inhibitory MMP-9–TIMP-1 complex revealed contacts between FBN and the TIMP-1 C-terminal domain. In the complex association study that shows no FBN effect, this TIMP-1 part was truncated to exclude any HPX binding^14^. According to our modelling results, FBN is significantly involved in complex formation. The models described above are the starting points for assembling more complex structures. Below, we present two of them.

**Table 1:** The most important interactions stabilising the MMP-9–TIMP-1 complexes.

TIMP-1	MMP-9	Type	Fragmentary complex	Terminal inhibitory complex	Circular heterohexamer
Cys1	Glu402	HB	+	+	−
Thr2	Leu188	HB, VdW	+	+	−
	Val398	VdW	+	+	
Cys3	Tyr423	HB	+	+	−
Val4	Leu188	VdW	+	+	−
	Tyr393	VdW	+	+	−
	Tyr423	VdW	+	+	−
Pro5	Met422	VdW	+	+	−
Pro6	Met422	VdW	+	+	−
Leu34	Tyr179	HB, VdW	+	+	−
	Pro180	VdW	+	+	−
Tyr35	Tyr179	VdW	+	+	−
Pro64	Tyr179	VdW	+	+	−
Ala65	Tyr179	VdW	+	+	−
Val69	Tyr179	VdW	+	+	−
	Phe192	VdW	+	+	−
Arg114	Glu82	Elect, HB	−	−	+
Leu133	Tyr393	VdW	+	+	−
	Tyr423	VdW	+	+	−
Ile135	Phe425	VdW	+	+	+/−
	Tyr696	VdW	+	+	+
	Tyr699	VdW	+	+	+
	Phe678	VdW	+/−	+/−	+
Pro136	Tyr696	VdW	+/−	+/−	+
	Tyr699	VdW	+	+	+
Cys137	Val694	2 × HB, VdW	+	+	+
Lys138	Asp390	Elect, HB	+	−	−
Leu139	Leu371	VdW	+	−	−
Leu146	Val694	VdW	+	+	+
Thr148	Val694	VdW	+	+	+
Leu151	Phe425	VdW	+	+	−
	Val694	VdW	−	+	−
Leu152	Phe678	VdW	+	+	+
	Val694	VdW	+	+	+
Glu156	Tyr420	HB	+	+	−
	Arg424	Elect, HB	+	+	−
Phe159	Pro658	VdW	+	+	+
	Leu659	VdW	+/−	+	−
	Phe678	VdW	+/−	+	+
	Trp680	VdW	+	+	+
Arg169	Glu63	Elect, HB	−	−	+
	Glu687	Elect, HB	−	−	+
Glu170	Arg685	Elect, HB	+	−	−
Trp176	Trp680	VdW	+	+	+
	Val691	VdW	+	+	+
	Val694	VdW	+	+	+
Ser178	Asp651	HB	+	+	+
Leu179	Leu72	VdW	−	−	+
Arg180	Glu63	Elect, HB	−	−	+
	Glu649	Elect, HB	+	+	−
	Glu687	Elect, HB	−	−	+
Ser181	Glu63	HB	−	−	+
	Arg652	HB	+/−	+/−	+/−
Ala184	Arg645	Elect, HB	+/−	+/−	+/−
	Arg652	Elect, HB	+/−	+/−	+/−

Hydrogen bonds (HB), Van der Waals (VdW), and Coulomb electrostatic interactions (Elect) are marked as follows: ‘+’ indicates stable interactions, ‘−’ the absent ones, and ‘+/−’ those that were repetitively formed and dissociated during MD.

The first is a terminal MMP-9–TIMP-1 inhibitory complex. It appears at the end of the MMP-9 enzymatic path—after secretion, activation, and finally inactivation by TIMP-1, which was already bound to its HPX. The second one is a circular-shaped model, which according to experimental data can be secreted by some cell types and might even not be supposed to undergo activation in physiological conditions, but to exhibit regulatory binding activity to free TIMP molecules. Its biological function, however, is not well established yet. In case of second model, as PRO is still present, the described TIMP-1 interface with CAT is unreachable, and new contacts are formed. TIMP-1 contacts with HPX are maintained in all types of complexes with little variety, resulting from the closest environment of the unstructured TIMP-1 loops. Because the presented models are subsets of the whole system, it is not obvious if all contacts are conserved in a system representing a full inhibitory complex. Also, in the case of the circular proMMP-9 trimeric model, the contacts require a more detailed investigation, because in different arrangements some parts of interfaces may differ. This is a reason to study the complete structures of those biological constructs.

### Model of the terminal MMP-9–TIMP-1 complex

**Figure 1:**
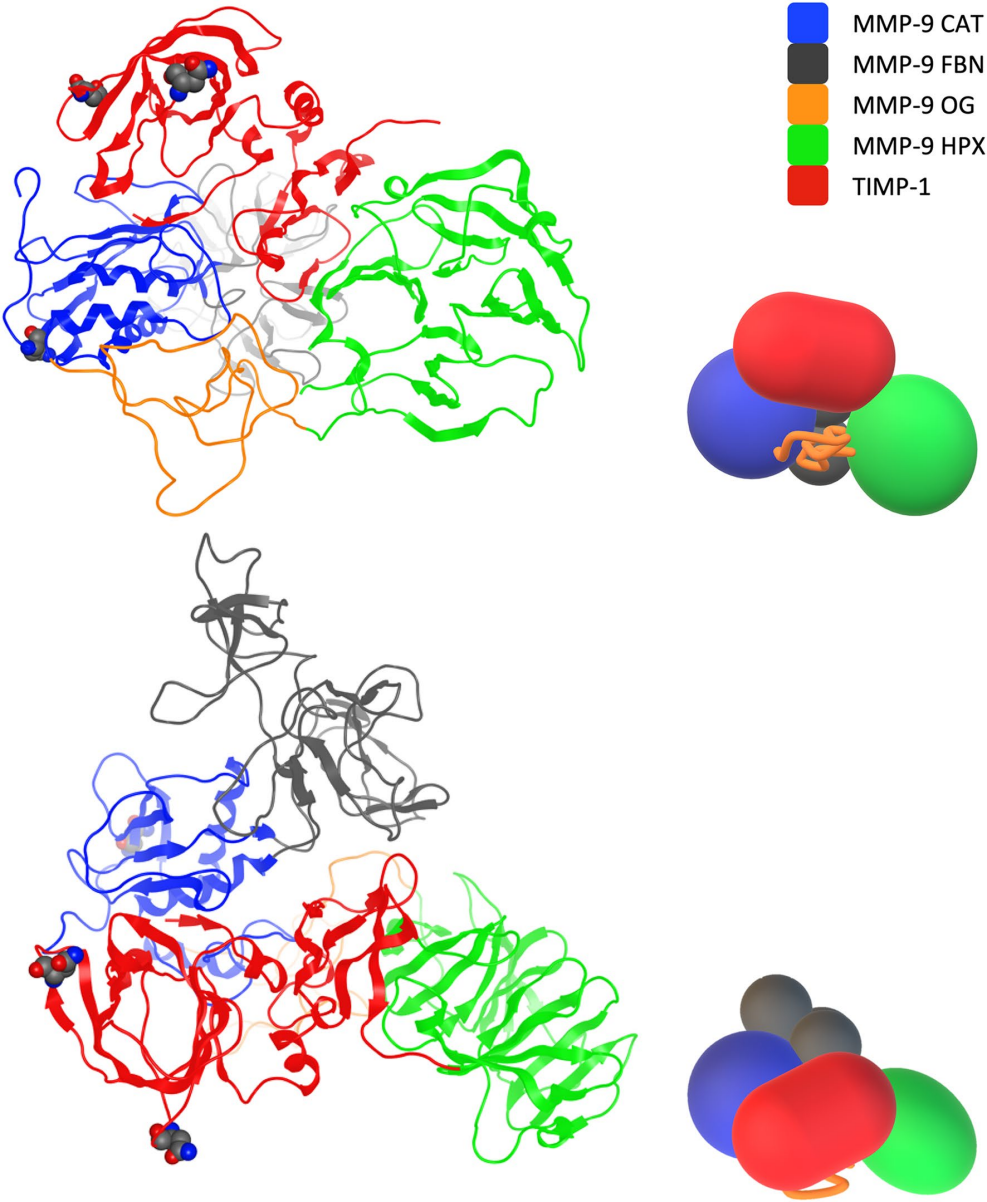
Structural model of the terminal-inhibitory MMP-9–TIMP-1 complex. On the left—a ribbon presentation of the structure; on the right—a schematic representation indicating existing domains and their mutual arrangement. Catalytic (blue), fibronectin (grey), OG (orange), and hemopexin (green) domains form a compact structure. TIMP-1 (red) forms contacts with the catalytic, fibronectin, and hemopexin domains. Amino acids undergoing N-glycosylation are shown as Van der Waals spheres. The bottom images are rotated 90 degrees around the horizontal axis, in relation to the top images.

The two created models of complexes were merged using TIMP-1 coordinates as the basis for structural alignment (Fig. 1). The resulting structure presented minor steric clashes between HPX and FBN. Due to the high mobility of FBN, also observed in our MD simulations, we decided to adjust it to the overall complex structure applying the energy minimisation. TIMP-1 134–142 residues, which in the fragmentary complex with CAT form an unstructured loop, significantly changed their positions due to the presence of HPX. They extended their structure to the IV blade of HPX. Therefore, in the terminal-inhibitory complex Lys138-Asp390 and Leu139-Leu371 interactions are missing (Table 1).

It should be noted that in the terminal-inhibitory complex, the Leu151-Val694 contact appears because, after binding of CAT, Phe425 pushes Leu151 towards HPX. In addition, FBN in the terminal-inhibitory complex breaks the interaction between Glu170 and Arg685. Additionally, Leu659 and Phe687 of MMP-9 interact with Phe159 of TIMP-1. This arrangement is mobile in the fragmentary complex, where Phe159 swaps its position between two poses. This is because the Leu151 sidechain is moved away from Phe687.

**Figure 2:**
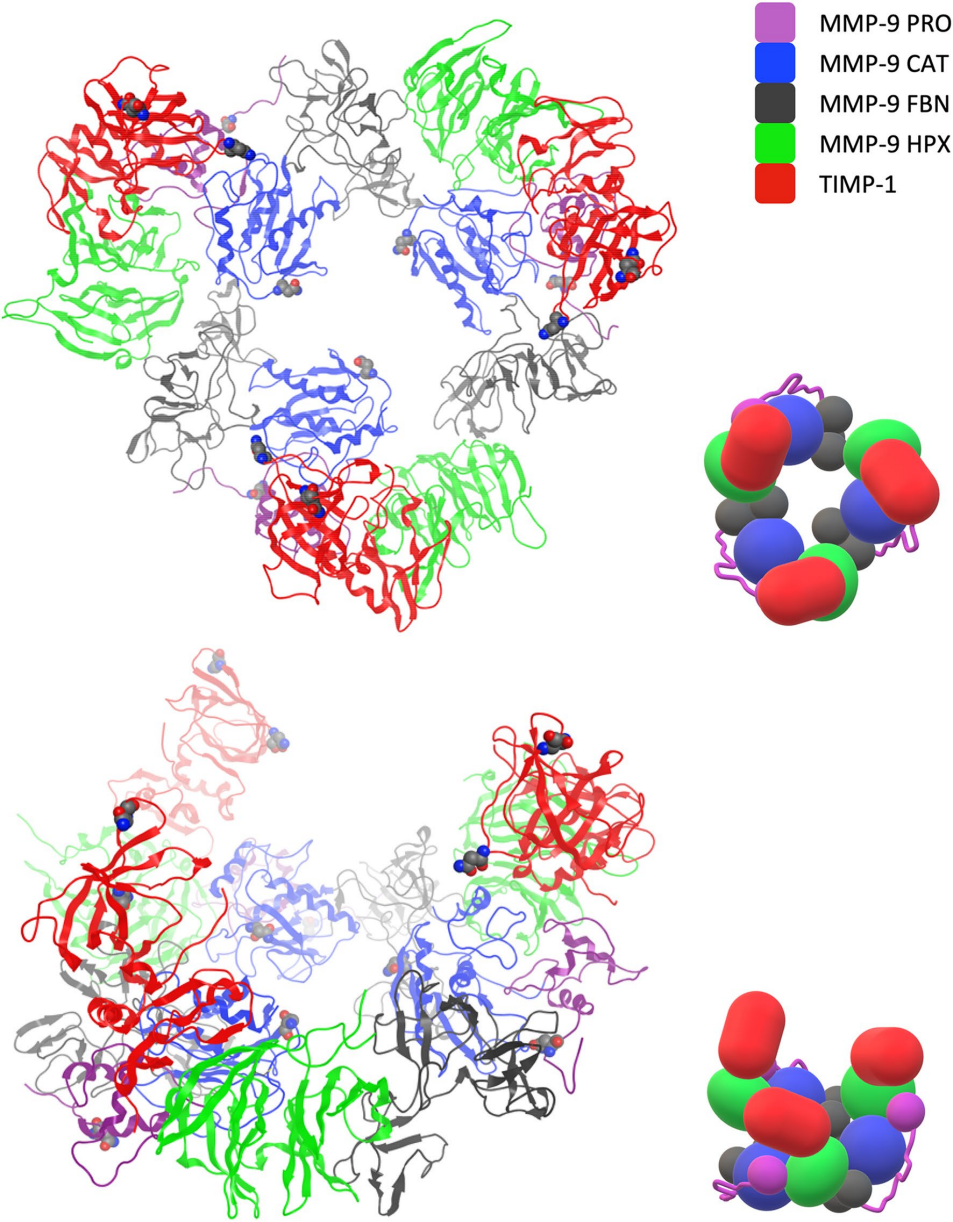
Structural model of the circular proMMP-9 heterohexamer with TIMP-1. On the left – a ribbon presentation of the structure; on the right – a schematic representation indicating existing domains and their mutual arrangement. The hemopexin domain (green) interacts with the catalytic (blue) domain, propeptide (purple) of one subunit, and with the fibronectin (grey) domain of the other one. TIMP-1 is attached to the complex in a direction that does not change the diameter of the complex. Amino acids undergoing N-glycosylation are shown as Van der Waals spheres. The bottom images are rotated 60 degrees about the horizontal axis, in relation to the upper images.

Furthermore, OGD was built as a straightened loop, and its stability was checked by applying MD. During 150 ns, its conformation folded into a lobe. We believe that such a compact structure refers to Rosenblum’s findings when modelling full-length monomeric MMP-9^32^. HPX, with the TIMP-1 molecule, can easily migrate through OGD, presenting such conformation. AFM measurements^18^ show that the OGD diameter in proMMP-9 is about 30 Å. Our results remain consistent with this description; however, it should be noted that HPX in the terminal-inhibitory MMP-9–TIMP-1 complex is rotated over the OGD lobe. Similarly to the presented OGD models, our structure, represented by a chain, is free from knots and is able extend in other biological processes^32^. The whole complex forms one stable compact structure, where the interaction energy between MMP-9 and TIMP-1 is − 150.09 ± 0.21 kcal/mol. The obtained OGD structure should be treated with some reserve because glycans were not introduced into the model. It was found that there are 14 possible O-glycosylation sites in OGD because they share two possible core glycans, namely Galβ1-3GalNAcα1-R and GlcNAcβ1-6[Galβ1-3]GalNAcα1-R, which are elongated in a number of ways^33^. In our model, all these sites are exposed to the solvent, which makes glycosylation possible (Fig. 2). Moreover, additional interactions between HPX and FBN were indicated (Table 2). In such a system, MMP-9 undergoes internalisation caused by the LRP-1 cargo receptor. It was found that, unlike MMP-2 /TIMP-2, the MMP-9 molecule is bound directly to LRP-1, and the binding site is localised on HPX at a site not overlapping with the TIMP-1 interface. The exact binding site, however, remains unknown^18^.

**Table 2:** Description of interactions between the MMP-9 domains.

PRO/CAT	HPX	Type	Terminal inhibitory complex	Circular heterohexamer
Glu53	Ala647	VdW	−	+
Tyr54	Arg652	HB, VdW	−	+
Arg56	Asp634	Elect, HB	−	+
	Arg645	VdW	−	+
	Ser646	VdW	−	+
Val57	Leu632	VdW	−	+
	Arg634	VdW	−	+
	Ser646	VdW	−	+
	Ser648	VdW	−	+
Glu59	Arg634	Elect, HB	−	+
Leu73	Glu649/Met653	VdW, VdW	−	+
	Asp652	VdW	−	+
Lys76	Glu649	Elect, HB	−	+
	Asp651	Elect, HB	−	+
Leu104	Ala647	VdW	−	+
Arg106	Asp609	Elect, HB	−	+
Phe110	Ala608	VdW	−	+
Gly112	Arg600	VdW	−	+
	Ala608	VdW	−	+
Asp113	Arg585/Arg600	Elect, HB/Elect, HB	−	+
Leu114	Ala608	VdW	−	+
	Asp609	VdW	−	+
Phe222	Gln586	HB	−	+
	Pro598	VdW	−	+
Asn224	Pro564	VdW	−	+
Arg249	Val595	VdW	−	+
	Gly597	HB	−	+
Asp251	Val595	VdW	−	+
Asp263	Arg546	Elect, HB	+	−
Thr264	Arg546	VdW	+	−
Asp266	Arg565	Elect, HB	−	+
Phe268	Pro561/Pro564	VdW/VdW	−	+
	Ala562	VdW	−	+
	Leu563	VdW	−	+
Phe270	Pro598	VdW	−	+
Glu427	Arg677	Elect, HB	+	−
	Tyr696	HB	+	−

Hydrogen bonds (HB), Van der Waals (VdW), and Coulomb electrostatic interactions (Elect) are marked as follows: ‘+’ indicates stable interactions and ‘−’ the absent ones.

TIMP-1, unlike TIMP-2, is expressed as an N-glycosylated protein. Under physiological conditions, the same mildly branched glycan is attached to the Asn30 and Asn78 amino acids^34^. Although glycosylation is not required for the binding process of metalloproteinases, the attached glycan molecule forms additional hydrogen bonds along with hydrophobic interactions. As shown for MMP-2, the glycan linked to Asn30 interacts with CAT, while Asn78 provides a larger contribution to the binding energy forming contacts with FBN and HPX^34^. The bi-antennary glycan can potentially be elongated by *N*-acetylglucosaminyltransferase-V, attaching β1,6-acetylglucosamine to its structure, which results in reduced inhibitor activity. Such an aberrantly glycosylated TIMP-1 is associated with the invasion of cancer cells and metastasis^35^. One should note that in the presented model, the glycosylated residues are located on the surface of the complex; therefore, the addition of glycan should not result in any steric conflicts with MMP-9.

### The model of the MMP-9–TIMP-1 heterohexamer and its biological implications

Protein–protein docking^26^ resulted in 11 structures that were selected as optimal for the circular homotrimeric structure assembly; 3 of them formed contacts with FBN and 8 with CAT. Successive, pair-wise overlaps of those interfaces revealed that only one option resulted in a purely circular-shaped structure, while helical or straight linear structures were excluded. In this arrangement, HPX forms contact with CAT through its III beta-propellers blade and with FBN through the II blade (Fig. 2). The structure appeared to be stable in MD. The most common contacts occurring at the interfaces were identified and are listed in Table 2.

The internal diameter of the homotrimer model is 24 Å, while the external diameter is 156 Å. Experimental estimates of dimensions obtained by Vandooren et al. for the smaller conformer are 28 ± 7 Å and 108 ± 21 Å for the internal and external diameter, respectively^15^. Our model’s internal diameter is in agreement with experimental data, and the external diameter is slightly larger than the one estimated using AFM. Despite this difference, we believe that our model is correct. This is because, firstly, such conformation allows the preservation of contacts of the C-terminal TIMP-1 domain with HPX of MMP-9. Secondly, TIMP-1 forms additional interactions with proMMP-9 CAT, which justifies its increased affinity to the trimer (Table 1)^15^.

Our trimeric model does not clarify the existence of a bond between Cys468 and Cys674. Since the former cysteine is located in a very flexible fragment, it is considered as being able to form a bridge; structural data show that the latter is not accessible for solvent. Therefore, most researchers describe homo-oligomers as dimers, where the bridging is carried out exclusively by Cys468^19^. OGD is indeed long enough to meet the geometric requirements, but it is uncertain whether the bridge is formed at all. Cys674 is buried inside the beta-propeller structure, inaccessible for bridging. However, Cys674 is located at the end of a beta-strand, with a beta-turn protruding from the overall propeller structure, which, although slightly bent towards TIMP-1, may expose the considered residue. This remains unclear, as is current knowledge on the MMP-9–NGAL complex, which is also stabilised by a covalent bond^36^. Due to the high importance of Cys516-Cys704 disulphide linking^16^, it is likely that Cys674 or Cys468 is involved in the NGAL binding^12^. However, the involvement of Cys674 in homotrimerization would indicate that such a structure is unable to bind NGAL. NGAL in such a dimer prevents the degradation of MMP-9; therefore, it positively regulates MMP-9 activity by stabilisation of the enzyme. This activity is correlated with poor prognosis in patients with breast cancer^37^.

It is not known exactly how the domains of individual proMMP-9 molecules are located in the homotrimer. We have built models of all three possible structures introducing OGD into the system to form the Cys468-Cys674 SS-bond to find which of them are possible. The models show that OGD is long enough to allow the formation of this disulphide bond with Cys674 belonging to any HPX. The lowest mean interaction energy of interaction between OGD and HPX, derived from MD simulations, corresponds to an arrangement where HPX is most distant to CAT/FBN of the same subunit. In this arrangement, the interaction energy is lower by about 18 kcal/mol, as compared to the two other options (− 34.03 ± 0.17 kcal/mol vs. − 14.03 ± 0.10 kcal/mol and − 17.77 ± 0.12 kcal/mol). Because the C-terminal part of OGD is highly extended in such an arrangement, we suggest that this would be impossible in the larger type of circular complex described by Vandooren et al.^15^ Because the homotrimers form two distinct populations differing in size, this particular feature may indicate the type of population to which the trimer belongs.

The Cys468-Cys674 bond formation allows another interaction between OGD and HPX. The sidechain of Pro469 enters into the hydrophobic region between the I and IV blade of HPX. Consequently, two groups of amino acids are moved closer to each other forming hydrogen bonds and Van der Waals interactions. These are Val467, Thr470, and Gly271 for OGD, and Asp660, Asp663, Asp676, and Arg677 for HPX. According to NMR and light scattering experiments carried out for the OSM protein, the O-glycosylation of the polypeptide chain reduces its flexibility and induces an extended conformation^38^. OGD contains 14 O-glycosylation sites^39^, so it is highly probable that it is extended. Such conformation is present in two models, where OGD links CAT with HPX, which is not the closest to it. In such conformations, a part of OGD, between CAT and Cys476, is highly extended. All potential glycosylation sites in those models are in positions in which glycan O-linking is possible. The stability of all models was confirmed with MD simulations. Additionally, such conformation would not interfere with glycans N-linked to Asn38 because this residue is positioned outside the circular structure. The N-glycosylated Asn120 is directed to the interior of the circle, and two options are possible. If the N-linked glycans are directed through the interior of the circle, all combinations of OGD linkage are possible. If not—probably only HPX, closest to the CAT C-terminus, can be linked to it. Interactions between the MMP-9 HPX and structured parts of TIMP-1 are mainly conserved in both modelled types of complexes. Slight differences in the interface result from a different sequence of events in protein processing (Table 1).

The creation of circular homotrimers is associated with a completely new orientation of PRO/CAT/FBN relative to HPX, hence the contact area is different (1297.79 ± 2.75 Å^2^). This interface does not resemble the terminal-inhibitory complex and can be divided into PRO/CAT and FBN parts. TIMP-1 interacts with HPX of MMP-9 through its IV beta-propeller blade. C-terminus conformational searching with LowModeMD provided two distinct results. Analysis of their MD trajectories suggests that one of them is more stable and likely, so only this one should be considered. The main interactions identified in the system are listed in Table 1. The mean interaction energy between all TIMP-1 molecules and MMP-9 domains was established as − 295.43 ± 0.48 kcal/mol. This, per unit, is significantly lower than in the fragmentary complex containing HPX, which can be considered representative for the non-inhibitory monomeric proMMP-9–TIMP-1 complex. However, the value per unit is higher than the value found in the terminal-inhibitory complex. These results suggest that the binding of TIMP-1 to homotrimeric proMMP-9 is preferable to its monomeric form, but still the terminal-inhibitory complex is the most energetically favourable. The internal energy of the C-terminal TIMP-1 in heterohexamer is − 46.76 ± 0.79 kcal/mol. Interactions stabilising the complex are listed in Table 1.

**Figure 3:**
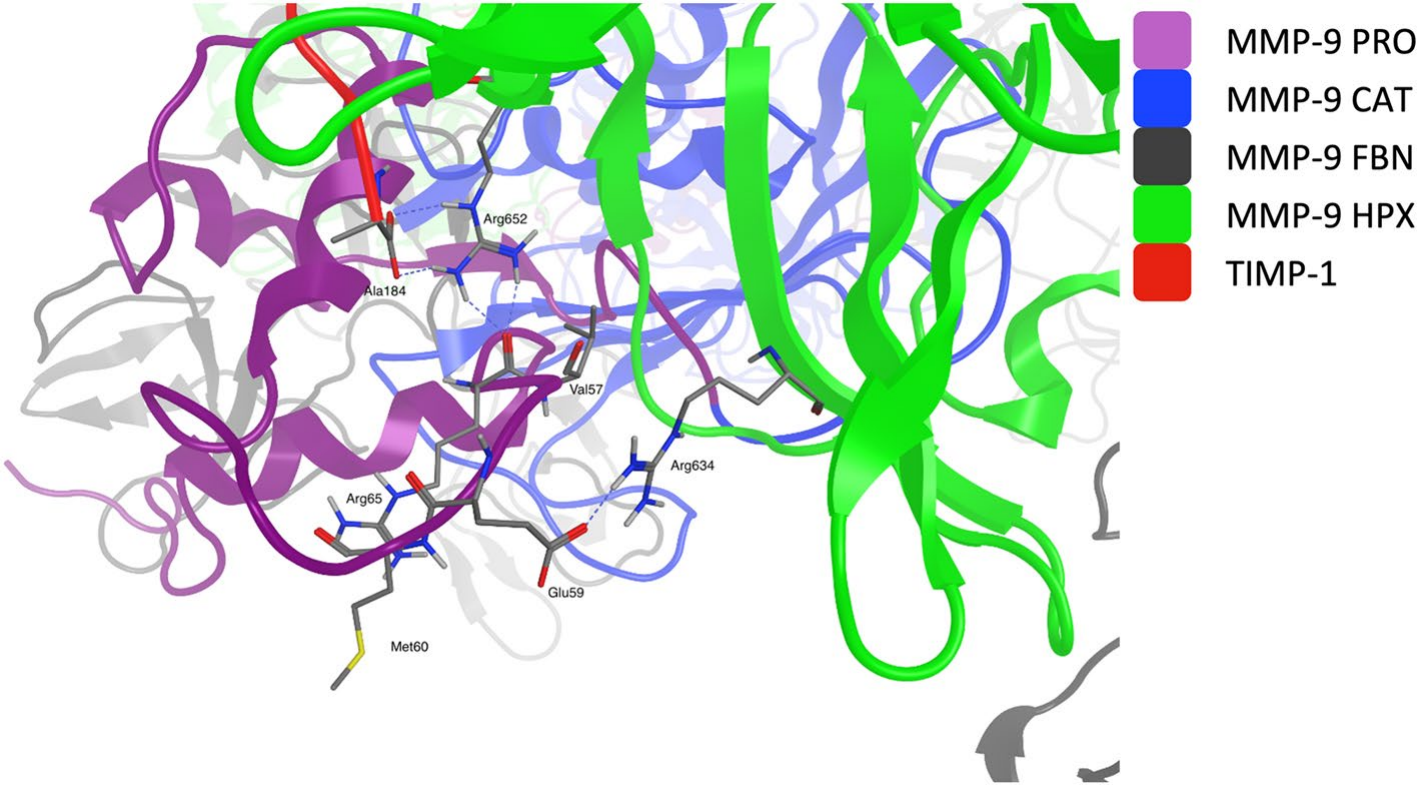
The proMMP-9 bait region in the circular heterohexamer. Propeptide (purple) Glu59 forms an electrostatic interaction with HPX (green) Arg634, which stiffens the bait region, making it less accessible for the MMP-3 molecule. The Val57 residue, which is supposed to fit into the MMP-3 S3 site during MMP-9 activation, is buried deep inside the structure of the MMP-9 homotrimer. TIMP-1 C-terminal Ala184 forms a double hydrogen bond and interacts electrostatically with Arg652 of the hemopexin domain.

In the circular heterohexamer, MMP-9 is still in a latent state, therefore TIMP-1 residues 1 to 69 cannot interact with its active site and with FBN, similarly to the fragmentary or terminal-inhibitory complexes (Fig. 2). HPX is oriented in a way that allows TIMP-1 binding in a very similar manner as in the terminal-inhibitory structure, with some differences related to the TIMP-1 C-terminus (residues 180 to 184), which in the circular heterohexamer curls up instead of extending parallel to the III hemopexin blade, as in the terminal-inhibitory or fragmentary complex, because this location is occupied by the PRO loop between helixes 1 and 2, i.e. Gly53-Glu63 residues (Fig. 3). This site, called the bait region, includes the initial cleavage region for MMP-3 (Glu59-Met60). Val57, which is supposed to be placed in the hydrophobic S3 pocket site of MMP-3, is buried in the structure and is inaccessible. The Glu59 residue forms an electrostatic interaction with Arg634, which limits the mobility and accessibility to the bait region, suggesting that the MMP-9 homotrimer is less susceptible to MMP-3 activation than the monomeric form. This phenomenon is consistent with the postulated function of the complex because the enhanced TIMP-1 binding prevents its association with the proMMP-9 monomers^15^. It is also consistent with experimental findings referring to the proMMP-9 homomultimeric form, which most likely has been mistakenly interpreted as a homodimer^17^. The same studies, however, reported a similar affinity of monomers and multimers to TIMP-1, which may suggest that the cited study was conducted using a different proMMP-9 form, or a mixture of forms.

An interesting shift in the interfaces is related to TIMP-1 Phe159, which in the circular heterohexamer sticks to Phe678 and breaks off contacts with Leu659. Also, Leu151 no longer interacts with Val694 due to the different positioning of CAT, which in the present model cannot link these residues together. Four new interactions between TIMP-1 and MMP-9 PRO have been found. Those are Arg114–Glu82, Arg169–Glu63, Leu179–Leu172, and Ser181–Glu63. Interestingly, MMP-9 Glu63 influences TIMP-1 Arg169 in such a way that it forms another interaction with Glu687.

TIMP functions are much more diverse than just inhibiting or regulating the MMP activation process. They play a role in promoting cell growth and suppressing excessive angiogenesis. They are also involved in programmed cell death. TIMP-1 and TIMP-2 revealed antiapoptotic properties in a number of cell lines, while TIMP-3 promoted this process. Interestingly, in T lymphoblastic lymphoma cell lines, TIMP-2 showed proapoptotic properties^40^. It acts similarly in the lymphocytes T cell line derived from peripheral blood, but only in the activated ones. TIMP-1 does not show such activity^41^. In this context, TIMPs cannot be treated solely as MMP activity regulators, and thus the enhanced TIMP-1 binding can contribute to the regulation of its bioavailability as a signalling molecule.

Neutrophil cells perform an additional proMMP-9 modification: they secrete N-terminally truncated MMP-9, lacking 8–10 amino acids^42^. It is worth noting that these cells are unable to synthesise MMP-2, as well as any of the TIMPs^43^.

## Conclusions

Molecular modelling methods were applied to build inhibitory and non-inhibitory MMP-9—TIMP-1 complexes, and a detailed description of these structures should contribute to a much better understanding of the MMP-9 regulatory processes. The modelling results are consistent with existing experimental data and observations, including TEM images. The structure of the most stable MMP-9–TIMP-1 complex (Figs. 2 and 3) should enable the design of new low-molecular-weight compounds that could help control MMP-9 activity. Molecules that can bind to the HPX and/or CAT/FBN domains can potentially stabilise such a complex, lowering its free energy of binding and affecting association/dissociation constants. It should be noted that the newly detected interface between these two domains can also be used to shift the equilibrium constant to achieve the required biological effect. In conclusion, understanding the structure of the MMP-9–TIMP-1 complex and its potential binding sites should significantly aid in the design of highly specific inhibitors, which is still a huge problem for MMP. This, in turn, should help to optimise therapeutic procedures against cancer and neurodegenerative diseases. The MMP-TIMP imbalance was shown to be associated with a wide variety of neoplasms, such as lung, colon, breast, and prostate cancers^44^. Peptidomimetic and small-molecule MMP inhibitors have already been used in clinical trials. However, different forms of MMP-9 can exhibit different sensitivities to some processing, as demonstrated by the inhibition of MMP-9 by alpha-2-macroglobulin^45^. Therefore, some inhibitors that are potent on MMP-9 monomers may not be suitable for trimer inhibition.

The role of MMPs in many lung diseases has been unquestioned for years^46^. As MMP-9 secretion promotes inflammation and destruction of lung tissue during lung injuries, it has been proposed that targeting MMP-9 activity could be beneficial in COVID-19, as well—especially because an increase in the MMP-9 level in circulating blood has been observed prior to respiratory failure in COVID-19 patients^47^. Moreover, in a meta-analysis study, MMP-9 has been established as a central point in the interactome network of melatonin and chloroquine in terms of immunoregulation in COVID-19^48^. Given the severity of these diseases, the properties and potential functions of circular trimeric MMP-9 in binding to TIMP-1 should not be underestimated.

Despite MMP-9 overactivation, it has been shown that free TIMP-1 molecules also promote cancer growth^49^. Therefore, it might prove beneficial for therapy to affect mostly TIMP-1 interaction with the trimeric population. In the last few years, the approach to specific blockers of protein–protein interfaces has been explored in therapy^50^. Applying such blockers to trimeric or monomeric interfaces only might shift the association constant of TIMP-1 in the desired manner, thus affecting its bioavailability for other molecules. In this case, however, further studies of differences in biological functions of MMP-9 forms are required.

Atomic coordinates of the modelled complexes are given in Supplementary Information, which enables the use of these structures by other research groups, and it allows them to be refined in future studies; for example, regarding the use of molecular replacement methods in X-ray and/or neutron diffraction techniques.

## Supplementary Information

Supplementary Information.

## Data Availability

Supplementary Information contains two files of coordinates (in Å) of the inhibitory and non-inhibitory MMP-9–TIMP-1 complexes and can be found in the online version of this article.
